# Vascular Endothelial Growth Factor: A Translational View in Oral Non-Communicable Diseases

**DOI:** 10.3390/biom11010085

**Published:** 2021-01-12

**Authors:** Sven Niklander, María José Bordagaray, Alejandra Fernández, Marcela Hernández

**Affiliations:** 1 Unit of Oral Pathology and Medicine, Faculty of Dentistry, Universidad Andres Bello, Viña del Mar 2520000, Chile; sven.niklander@unab.cl; 2 Laboratory of Periodontal Biology, Faculty of Dentistry, Universidad de Chile, Santiago 8380544, Chile; mbordagaray@odontologia.uchile.cl (M.J.B.); alejandra.fernandez@unab.cl (A.F.); 3 Department of Conservative Dentistry, Faculty of Dentistry, Universidad de Chile, Santiago 8380544, Chile; 4 Unit of Oral Pathology and Medicine, Faculty of Dentistry, Universidad Andres Bello, Santiago 8320000, Chile; 5 Department of Oral Pathology and Medicine, Faculty of Dentistry, Universidad de Chile, Santiago 8380544, Chile

**Keywords:** VEGF, VEGFR, angiogenesis, periodontitis, periimplantitis, apical periodontitis, oral cancer, oral squamous cell carcinoma

## Abstract

Vascular endothelial growth factors (VEGFs) are vital regulators of angiogenesis that are expressed in response to soluble mediators, such as cytokines and growth factors. Their physiologic functions include blood vessel formation, regulation of vascular permeability, stem cell and monocyte/macrophage recruitment and maintenance of bone homeostasis and repair. In addition, angiogenesis plays a pivotal role in chronic pathologic conditions, such as tumorigenesis, inflammatory immune diseases and bone loss. According to their prevalence, morbidity and mortality, inflammatory diseases affecting periodontal tissues and oral cancer are relevant non-communicable diseases. Whereas oral squamous cell carcinoma (OSCC) is considered one of the most common cancers worldwide, destructive inflammatory periodontal diseases, on the other hand, are amongst the most prevalent chronic inflammatory conditions affecting humans and also represent the main cause of tooth loss in adults. In the recent years, while knowledge regarding the role of VEGF signaling in common oral diseases is expanding, new potential translational applications emerge. In the present narrative review we aim to explore the role of VEGF signaling in oral cancer and destructive periodontal inflammatory diseases, with emphasis in its translational applications as potential biomarkers and therapeutic targets.

## 1. Introduction

Vasculogenesis, the formation of blood vessels from de novo generation of endothelial cells, and angiogenesis, the process of new blood vessel formation, are critical during development and physiologic homeostasis, but can also mediate the pathogenesis of several diseases. Vascular endothelial growth factors (VEGFs) are vital regulators of angiogenesis and vasculogenesis that are expressed in response to soluble mediators, such as cytokines and growth factors [1]. The physiologic functions of the VEGF signaling axis involve blood vessel formation-endothelial cell proliferation, migration, and cell survival-, regulation of vascular permeability and maintenance of bone homeostasis and repair, affecting the differentiation and function of osteoblasts and osteoclasts [2]. VEGF is also required for stem cell and monocyte/macrophage recruitment, maintenance of tissue barrier functions and neuroprotection [3]. In addition to these physiologic processes, angiogenesis plays a pivotal role in oral chronic pathologic conditions, such as tumorigenesis and inflammatory-immune diseases with bone loss [3,4]. 

VEGF is a type of glycoprotein, which possesses angiogenic, mitogenic and vascular permeability regulating properties, thus enhancing the activity of endothelial cells. The VEGF family includes VEGF-A, -B, -C and -D, as well as placental growth factor, which interact differently with cell-surface tyrosine kinase receptors, VEGFR 1–3. Overall, they regulate blood vessel morphogenesis and permeability, though VEGF-C and VEGF-D are primarily implicated in regulation of lymphogenesis [2]. As the prototypical VEGF, VEGF-A is considered the most potent stimulator of vasculogenesis and angiogenesis. In addition to increase vascular permeability, vasodilatation, and the recruitment of inflammatory cells, VEGF triggers the inhibition of apoptosis and increases cellular proliferation. Binding of VEGFR to its ligands, considered to be the “canonical signaling”, induces receptor homodimerization or heterodimerization, leading to activation of the tyrosine kinase and autophosphorylation of tyrosine residues in the receptor intracellular domains to initiate consecutive intracellular signaling pathways, including the PI3K and p38 MAPK pathways. This represents most probably the prevailing mechanism by which VEGF exerts its effects over proliferation, migration and vascular morphogenesis [5]. Alternatively, ligand-independent receptor activation or “non-canonical VEGF signaling” might be initiated i.e., by Src-mediated activation and downstream ligand-mediated trans-phosphorylation [1,6]. 

VEGF receptors are located in endothelial cells, but also in many non-endothelial cells, and act through autocrine pathways to regulate cell survival and function. The VEGFR1 gene codifies for two variants of the VEGFR1 receptor: a full-length VEGFR1/Flt-1 receptor with tyrosine kinase mRNA form; and a soluble form carrying only the ligand-binding region (sFlt-1/soluble VEGFR1) that functions as a decoy receptor by trapping its ligands VEGF-A, PlGF, and VEGF-B. This way gene expression is regulated through the balance between its full-length and soluble forms. Downstream activation of diverse pathways including phospholipase C-ɣ, protein kinase C, Ca^2+^, extracellular-signal-regulated protein kinase (ERK), Akt, Src, focal adhesion kinase and calcineurin have been implicated in mediating the multiple VEGF functions [5]. Moreover, there is a strong crosstalk between cell responses to hypoxia, cancer and inflammation. Both diseases create hypoxic conditions at the local site due to increased metabolic activity outpacing the availability of oxygen. Hypoxia inducible factor (HIF) is a pivotal transcription factor induced under hypoxia that transactivates target genes, including VEGF, and has a direct effect on the master switch of inflammation, nuclear factor (NF)-κB pathway. 

Accordingly, the coordinated output from these signaling systems controls angiogenesis, blood flow, tissue perfusion, inflammatory cell extravasation, and bone remodeling and repair [2,3]. Herein, we aim to explore the role of VEGF signaling in oral cancer and destructive periodontal inflammatory diseases, with emphasis in its translational applications as potential biomarkers and therapeutic targets. For this purpose, we analyzed the available literature using the following MESH terms: “VEGF”, “periodontitis”, “peri-Implantitis”, “periapical periodontitis” and “mouth neoplasm”. The studies used for this review included analytical and interventional original research articles and systematic reviews with or without meta-analysis available in English with focus on the last 10 years. 

## 2. Oral Cancer

Oral cancer is considered one of the most common cancers worldwide with a global incidence of more than 350,000 new cases and 177,000 deaths every year, with considerable geographic variations [7]. Ninety percent of all oral cancers are oral squamous cell carcinomas (OSCC), the most common form of oral cancer. The remaining 10% consist of salivary gland cancers, lymphomas, sarcomas and metastasis [8]. The overall survival rate of oral cancer is ≈50% during the first 5 years [9], mainly because most cases are diagnosed at advanced stages of the disease (stages III or IV) [10]. Many OSCCs develop from oral potentially malignant disorders (OPMDs) (lesions in which cancer is more likely to arise that can have different degrees of dysplasia), but only 5% of all OPMDs undergo malignant transformation [11]. 

Like normal tissues, tumors need nutrition, oxygenation and a system to evacuate metabolic wastes and carbon dioxide, which is granted by the tumor-associated neovasculature generated by the process of angiogenesis. Angiogenesis is considered a hallmark of cancer, as is essential for the growth, invasion and metastasis of tumors [12]. Neoplastic cells can only form a clinically observable tumor if the host is able to provide a vascular network. Tumors will not grow more than 1–2 mm in size unless an intra-tumoral capillary network is developed [13]. 

**Figure 1:**
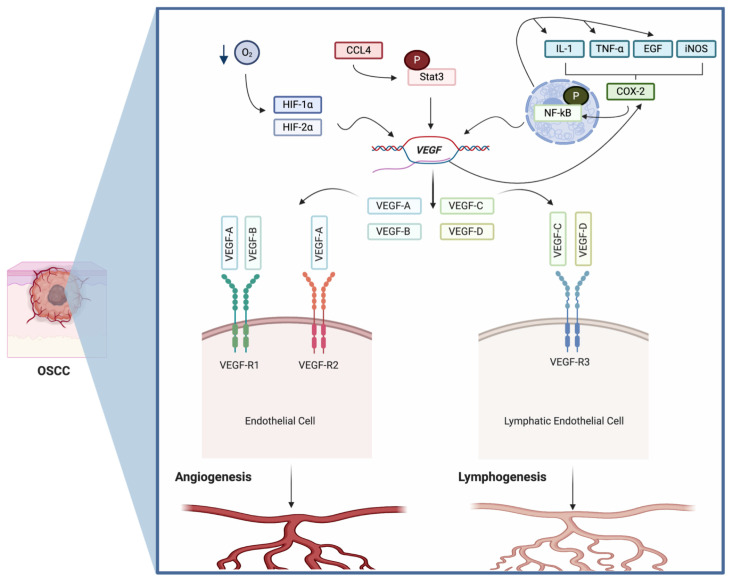
Role of VEGF signaling in OSCC. VEGF is constitutively expressed in oral squamous cell carcinoma. Its secretion can be the result of the activation of different signaling pathways, which includes: (1) the production of hypoxia inducible and HIF-2α by low oxygen levels, (2) the activation of Stat3 by CCL4 (which is constitutively expressed in OSCC) and (3) the activation of COX2 by different inflammatory molecules (commonly overexpressed in OSCC) which activate the NF-kB pathway. The activation of these pathways leads to the transcription of all four variants of VEGF (A–D), which through the activation of VEGF-R1, VEGF-R2 and VEGF-3 on blood and lymphatic endothelial cells, induces angio- and lymphogenesis. Created with BioRender.com.

VEGF is probably the most essential angiogenic factor expressed in cancer, as it plays a central role in regulating angiogenesis in solid tumors [1]. Animal models have shown that rapid tumor growth and microvascular density (MVD) are directly associated with VEGF expression [14], which is associated with the angiogenic switch. The angiogenic switch reveals the capability of neoplastic and inflammatory cells to produce angiogenic factors into the tumor microenvironment to stimulate proliferation and migration of endothelial cells to form a newly vasculature that provides oxygen and nutrients to the tumor [15]. As mentioned early, VEGF also participates in the recruitment of inflammatory cells and inhibits endothelial cell apoptosis [16] which are important features for the maintenance of the tumor neovasculature. VEGF helps in the recruitment of different inflammatory cells by inducing the activation of cyclooxygenase 2 (COX-2), which leads to NF-kB activation with subsequent release of inflammatory cytokines such as IL-1 and TNF-α, among others [17] (Figure 1). VEGF can inhibit endothelial cell apoptosis whether by inducing the expression of anti-apoptotic proteins, such as Bcl-2, survivin and A1, or by activating thePI3K/Akt pathway [18]. This promotes cell survival, by blocking the pro-apoptotic effects of BAD and Bax, and by inducing the expression of pro-survival genes (e.g., NF-kB) [19]. 

Originally it was thought that angiogenesis was only important for the rapid growth of macroscopically detected tumors. Nevertheless, it was later demonstrated that angiogenesis also contributes to the premalignant phase of neoplastic progression [12], which is also the case in OPMDs. In normal oral mucosa (NOM) VEGF is not usually detected or observed only in up to 30% of NOM samples with classical immunohistochemistry techniques, usually confined to the basal and parabasal layers of the epithelium [20]. Sauter et al. were the first to demonstrate a gradual and progressive increase of VEGF expression during the whole process of oral carcinogenesis, with weak or absent staining in NOM, moderate staining in moderate dysplasia, carcinoma in situ and early primary OSCC, and intense staining in advanced primary and metastatic OSCC [14]. 

In OPMDs, VEGF is diffusely expressed across the epithelium in 63% to 100% of all studied samples [21], whereas in OSCC it is usually expressed in 100% of the samples [22,23]. Mean MVD is also reported to be higher in OPMDs than in NOM [14,21] and has been positively correlated with the degree of dysplasia [15,21,24] and VEGF expression [20,25]. The degree of dysplasia has also been significantly correlated with VEGF and mast cell density (MCD) which are thought to be important for the initial stages of oral carcinogenesis as they contribute to angiogenesis [24]. 

There is robust evidence showing VEGF upregulation in oral cancer [14,21,24,26,27]. In OSCC, VEGF expression has been associated with tumor differentiation [26,28], clinical stage [29], nodal metastasis [21,30], distant metastasis [31] and overall survival [32]. Same as with oral dysplasia, mean MVD and MCD increase significantly in OSCC compared to NOM and have been associated with VEGF levels [24,25]. Mean MVD has also been positively associated with tumor size [30]. All VEGF family members (A–D) are expressed in OSCC. VEGF-A and VEGF-B are associated with angiogenesis, as their increase is correlated with an increase in mean MVD, whereas VEGF-C and VEGF-D are associated with lymph node involvement [33]. Expression of VEGF receptors (VEGFR1-3) has also been recently studied in OSCC. Using immunohistochemistry, it was found that 88% (44 out of 50 cases) of OSSCs overexpressed some form of VEGF-R. VEGFR-1 was overexpressed in 56% of the samples, VEGFR-2 in 42% and VEGFR-3 in 60%, but many samples expressed a combination of more than one variant. More importantly, VEGFR expression was associated with clinical parameters, such as neck node involvement, tumor size and tumor associated death [34]. 

The VEGF gene is considered a highly polymorphic gene with multiple single nucleotide polymorphisms (SNPs) which have been related with OSCC development and progression. VEGF-C rs766413 and rs20446463 polymorphism have been linked with oral cancer susceptibility in a Taiwanese population [35]. VEGF-A + 936 CC polymorphism has been associated with advanced OSCC and VEGF-A -1154 GG genotype is considered as an independent adverse factor for survival of OSCC patients [36]. A recent systematic review concluded that VEGF + 936 CT or TT polymorphism may be associated with an increased risk of oral cancer among caucasians [37]. 

It is well known that VEGF production and tumor angiogenesis are regulated by the interaction of multiple molecules. The increase in VEGF expression in oral cancer might be a response to tumor-associated hypoxia, as in vivo and in vitro studies have shown VEGF upregulation in decreasing concentrations of oxygen [30,38]. Hypoxia-inducible factor-1α (HIF-1α) is a transcription factor usually upregulated under hypoxic conditions and cumulative genetic alterations. In OSCC cells, HIF-1α expression is also regulated by the hepatoma-derived growth factor (HDGF), as its inhibition reduces HIF-1α and VEGF expression [39]. HIF-1α binds to hypoxia response elements and regulates changes in the expression of different factors, such as VEGF [40], plasminogen activator inhibitor-1 (PAI-1) and carbonic anhydrase 9 (CAIX) [28], promoting neovascularization and favoring tumor spread [41]. HIF-1α and HIF-2α have shown positive correlation with clinical-pathological parameters in OSCC, tumor size and MVD, and their knock down inhibited tumor angiogenesis and tumor growth in a nude mice xenograft model [42]. 

Although hypoxia contributes to the angiogenic switch, it is not the only mechanism involved. Signal transducers and activators of transcription 3 (Stat3) are considered an important regulator of VEGF expression in cancer. Stat3 regulates VEGF production through a putative Stat3 responsive element on the VEGF promoter, inducing VEGF mRNA transcription [43]. Stat3 is constitutively expressed in OSCC and its phosphorylation is associated with a more aggressive phenotype of the disease. An increase in its phosphorylated form was significantly correlated with VEGF production and intratumoral MVD [44]. COX-2 is another important regulator of VEGF in OSCC. COX-2 was demonstrated to regulate VEGF-C levels in OSCC in vitro [45] and in vivo [32,46]. More importantly, COX-2/VEGF-C co-expression is correlated with lymphangiogenesis, lymph node metastasis, TNM stage, lymphatic vessel density and is reported as an independent factor for survival [32]. A summary of the role of VEGF signaling in OSCC is presented in Figure 1.

### 2.1. Diagnostics

About one third of oral cancer patients develop a recurrent tumor after initial treatment. There are several biomarkers that have been proposed to be used for early diagnosis or as prognostic factors in OSCC, being VEGF one of them. Concomitant expression of VEGF and matrix metalloproteinase (MMP)-11, a MMP related with cancer cell survival, has been reported as a predictor for progression from precancerous stage to malignancy [25] and serum VEGF-A levels are reported to be higher in patients with OPMDs than healthy controls, with sensitivity and specificity values of 63% and 80% [21]. These data suggest VEGF as a possible biomarker for OPMDs. Nevertheless, there are limited reports that have investigated the potential of VEGF in predicting malignant transformation of OPMDs.

Serum VEGF levels are significantly higher in OSCC than in control patients [21,27,47] and high VEGF levels have been associated with late stage, large tumors and lymph node involvement [27]. According to this, Aggarwal et al. reported sensitivity and specificity values of 65.71% and 66.67% for distinguishing OSCC patients from controls [27]. Specifically, serum VEGF-A levels have also been found higher in patients with OSCC than healthy controls. Sensitivity and specificity values were 73% and 100%, respectively [21]. A follow-up study of 144 patients with OSCC for 115 months showed a direct correlation between VEGF levels and disease-free survival, concluding VEGF expression is an adverse prognosticator for disease-free survival [25]. Similar results have been reported by others [31,39,46,48,49]. VEGF expression has also been reported to improve accuracy and efficacy of prognostic prediction of OSCC. A strong VEGF-A or VEGF-C expression contributed to the histopathological diagnosis of vascular invasion, and histopathological feature associated with poor prognosis [22]. Recently, a meta-analysis that evaluated more than 180 biomarkers for oral tongue squamous cell carcinoma (OTSCC) concluded VEGF-A to be a useful prognosticator. Nonetheless, the authors concluded that although VEGF is a very promising biomarker, the utility of VEGF as a prognostic biomarker has to be evaluated in multicentre studies using large cohort of OTSCC samples following REMARK (Reporting Recommendations for Tumor Marker Prognostic Studies) criteria [50]. 

From all VEGF variants, VEGF-C and VEGF-D seem to be of particular importance for the development of lymph node metastasis. Upregulation of VEGF-C promotes peritumoral lymphangiogenesis and is associated with lymph node metastasis and poor 5-years disease-free survival [46]. High VEGF-C expression in primary tumors has been associated with a greater probability for the occurrence of micrometastases and isolated tumor cells in pathological staged N0 OSCC [51]. Also, VEGF-C is reported as an independent prognostic factor for lymph node metastases in early tongue cancer [52] and of OSCC survival [32]. In T2 or T3 cN0 OSCC patients, VEGF-D expression has also been associated with the presence of lymph node metastasis [53]. Because of the aforementioned, the expression of VEGF-C and VEGF-D are proposed as potential biomarkers for detecting and predicting lymph node metastases in OSCC. Nevertheless, other studies have failed in showing independent prognostic utility of VEGF-C for predicting risk of lymph node metastases, although they did find a significant association [54,55]. A synthesis of the studies conducted in diagnostics of oral cancer is presented in Table 1.

### 2.2. Therapy and Projections

In comparison to normal tissues, tumor vasculature presents atypical morphological features, such as dilated, tortuous and disorganized blood vessels. This leads to excessive permeability, poor perfusion, hypoxia, decreased immune cell infiltration and predisposition to metastatic dissemination [68], features that can impact negatively on clinical outcome. The development of an abnormal tumor vasculature is associated with an increase in different growth factors, of which VEGF is a key player [69]. VEGF mRNA is overexpressed in the majority of human tumors and correlates with invasiveness, vascular density, metastasis, recurrences, and prognosis. Thus, different strategies to inhibit the VEGF/VEGFR signaling pathway have been developed [1]. Angiogenesis inhibition by targeting VEGF has shown to be an effective treatment of OSCC in in vivo animal models [70,71,72] and in vitro [73]. There are several anti-VEGF family agents, which include bevacizumab, sorafenib, vandetanib, among others [74], that have been tested for OSCC treatment.

Bevacizumab, a humanized monoclonal antibody against VEGF-A, is one of the most commonly used drugs in oncology. It has FDA approval for the treatment of colorectal cancer, renal cell carcinoma, non-small-cell lung carcinoma, glioblastoma multiforme, ovarian cancer and cervical cancer [75]. Different phase II clinical trials have reported the utility of bevacizumab for treatment of squamous cell carcinoma of the head and neck (SSCHN)—which includes OSCC—as part of combinatorial treatments. This includes the use of bevacizumab in combination with: erlotinib [76], pemetrexed [77], cetuximab, cisplatin and concurrent intensity modulated radiation therapy [78]. A recent phase III clinical trial which evaluated the addition of bevacizumab to platinum-based chemotherapy in recurrent or metastatic SCCHN, showed that the addition of bevacizumab did not improve overall survival but did improve response rate and progression-free survival. Nevertheless, there was a significant increase in toxicity, including bleeding events and treatment related deaths [79]. 

Sorafenib and vandetanib are multi-kinases inhibitors that among their different targets, they also inhibit VEGFR. Sorafenib has FDA approval for the treatment of hepatocellular carcinoma [74] and in vitro studies have suggested promising effects of sorafenib as treatment agent for SCCHN [80,81]. Nonetheless, phase II clinical trials have shown modest [82,83] or none [84] anti-tumor activity of sorafenib for the treatment of recurrent or metastatic SCCHN. Vandetanib has also shown promising results in animal models for OSCC treatment. In a mouse 4-NQO model of oral carcinogenesis, vandetanib decreased the occurrence of tumors and dysplasia by reducing angiogenesis and proliferation, probably by inhibiting VEGFR and epidermal growth factor receptor (EGFR) [85]. Nevertheless, a phase II clinical trial that evaluated vandetanib in combination with docetaxel for the treatment of recurrent or metastatic SCCHNC showed limited utility of the proposed treatment regimen [86]. A synthesis of the studies conducted in therapeutics of oral cancer is presented in Table 2.

## 3. Inflammatory Diseases Affecting Periodontal Tissues 

Inflammatory conditions affecting periodontal tissues include periodontitis, peri-implantitis and apical periodontitis (AP). They share common etiopathogenic mechanisms that lead to the development of inflammatory periodontal lesions in response to oral bacterial consortia with alveolar bone loss as their main hallmark. While periodontal lesions in periodontitis and peri-implantitis comprise the marginal periodontal supporting tissues, the target of apical periodontitis are the peri-radicular periodontal tissues [89,90,91]. In this context, the striking role of VEGF in angiogenesis might relate to the formation of granulation tissue in destructive periodontal lesions [92], nutrient level restoration and immune cell migration [71]. Moreover, an intimate connection between immune cells and the endothelium occurs during inflammation. Immune cells induce the activation of the inflammasome and the NFκB signaling pathway, which in turn activates the VEGF/VEGFR axis in endothelial cells, inducing vasodilatation (edema) and increasing vascular permeability [21,22]. The activation of the endothelium permits leukocytes to transmigrate from the blood to the site of injury [40]. VEGF is the main soluble factor that modifies the endothelial barrier [41,42,43] and secreted by neutrophils, platelets, macrophages, activated-T cells, dendritic cells, pericytes, and the endothelial cells themselves [44].

### 3.1. Periodontitis and Peri-Implantitis

Periodontitis is a chronic immune-inflammatory disease that develops from the combination of a dysbiotic polymicrobial community and a susceptible host [93,94] resulting in the loss of periodontal supporting tissues. Its features include gingival inflammation and bleeding, pocket formation (pathologically deepened gingival sulcus), attachment loss and alveolar bone resorption [89,95]. Periodontitis is highly prevalent in adults and a primary cause of tooth loss. In addition, emerging evidence associates periodontitis with higher morbidity and mortality of systemic non-communicable diseases, such as cardiovascular diseases [94,96]. On the other hand, peri-implantitis is the inflammation of the adjacent soft and hard tissues surrounding a dental implant. In a similar manner to periodontitis, it is clinically characterized by bleeding, increased pocket formation and suppuration, resulting from polymicrobial anaerobic infection that leads to rapid bone resorption and implant failure [91,97,98]. 

Periodontitis is characterized by angiogenic changes within the periodontal tissues. These include the neoformation of loop-like blood vessels in association with increased vascular permeability. These changes appear to facilitate the arrival of proinflammatory cells, chemical mediators, and growth factors to the periodontal tissues, which ultimately exacerbate periodontal inflammation and destruction [99,100,101]. VEGF has been highly expressed in gingival tissue samples from periodontitis patients in relation to gingivitis and healthy gingiva [102,103]. VEGF has been detected in the depths of the gingival stroma, particularly within smooth muscle cells of vascular structures, macrophages, mast cells, fibroblast-like cells, neutrophils and plasma cells in addition to endothelial cells [93,103]. Up to now, few reports have explored an association between VEGF and SNPs in periodontitis. Specifically, the position-936 has been previously associated with periodontitis [104] whereas 2578 C/A, rs699947 polymorphism remains controversial [105]. In the same line, higher VEGF immunohistochemical expression in the mucosa of peri-implantitis patients was reported in comparison to peri-implant healthy mucosa and peri-implant mucositis, in which no hard tissue loss occurs [106]. In contrast, lower expression of VEGF was observed in soft tissues surrounding failing implants than in normal gingiva [107]. These findings suggest that VEGF may play a role in the pathogenesis and/or progression of periodontitis and peri-implantitis.

Also, overexpression of VEGF-C in transgenic keratin 14 (K14)-VEGFC mice has been reported, which was followed by lymphatic vessel hyperplasia in normal gingival tissues, without changes in the blood vessels. In normal conditions, K14-VEGFC mice showed increased recruitment of immune cells and higher alveolar bone in relation to their wild-type littermates. Nevertheless, after induction of periodontitis, K14-VEGFC and wild-type mice showed no significant differences in bone resorption, angiogenesis, recruitment of immune cells, levels of MMPs, proinflammatory cytokines, and bone-related proteins in gingival tissue samples. Therefore, VEGF-C may participate in lymphatic endothelial cell proliferation with no impact in periodontitis development [108]. VEGF-C, -D, and VEGFR-3 have been detected in endothelial cells and keratinocytes in healthy gingiva. VEGF-C and VEGFR-3 were also detected in fibroblast-like cells and lymphatic vessels, respectively. After challenging with lipopolysaccharide (LPS) or IL-6/sIL-6R complex, gingival fibroblasts increased the secretion and gene expression of VEGF-A and -C compared to non-induced controls, while VEGF-D was not detected. Altogether, VEGF-A and -C contributes to both the angiogenesis and lymphangiogenesis processes in the pathogenesis of periodontitis [109]. 

During periodontal inflammation, tissue damage increases oxygen consumption resulting in a hypoxic microenvironment. In this context, in vitro studies support the involvement of the HIF-1α. In line with this, a hypoxic microenvironment combined with the presence of lipopolysaccharide (LPS) from *P. gingivalis* -a keystone periodontopathogen in periodontitis- induce the translocation of HIF-1α into the nucleus, which then dimerizes with HIF-1β, up-regulating the transcription of VEGF in human periodontal ligament and gingival fibroblast cells [110,111,112]. In addition, IL-1 upregulated the transcriptional levels of HIF-1α in human gingival fibroblasts [113], which is the most abundant cell type in gingival connective tissues [114]. Accordingly, higher HIF-1α and VEGF concentrations have been found in gingival biopsies from periodontal pockets compared to gingivitis and healthy gingiva sites [115]. Likewise, other studies have shown that VEGF concentrations correlate positively with saliva and GCF HIF-1α concentrations in periodontal patients [57]. 

It is known that redox balance can modify periodontal tissue responses [113,116]. Recently, our research group showed that hydrogen peroxide treatment increased the levels of gelatinolytic MMPs activity in human periodontal ligament fibroblasts, through the activation of the NF-κB pathway and intracellular calcium signaling [117]. Hydrogen peroxide also upregulated VEGF and cytokine levels (including IL-6 and CXCL12), while MMP inhibition reduced the bioavailability of VEGF and CXCL12, also decreasing fibroblast migration and wound healing [117]. These results might be explained by atypical activation of the NF-κB pathway in response to the phosphorylation of IκBα and p65 caused by hydrogen peroxide [118,119]. The redox-MMP interaction highlights the complexity of the networks involving VEGF during periodontal inflammation. Arguably, VEGF bioavailability might result from proteolytic cleavage and release from its cryptic form from the extracellular matrix [117]. 

Evidence has shown the link between periodontitis and systemic diseases, including diabetes, cardiovascular disease, rheumatoid arthritis, and adverse pregnancy outcomes [94,120,121,122,123]. VEGF expression has been analyzed in both, periodontal tissue samples and oral gingival crevicular fluid (GCF) of diabetic patients to explore the plausibility of this association [124,125,126,127,128]. A recent review described higher VEGF expression and concentrations in periodontal tissue and GCF samples, respectively of diabetic patients with periodontitis compared to nondiabetic controls [128]. These outcomes suggest that diabetes might influence the expression of VEGF in periodontal tissues. It is theorized that this may be a consequence of insulin resistance and endothelial dysfunction, both frequently observed in patients with diabetes. In contrast, similar studies exploring cardiovascular disease show that the concentrations of VEGF in the serum and GCF of patients are unrelated to periodontitis [129,130]. 

### 3.2. Apical Periodontitis

Apical periodontitis (AP) is an immune-inflammatory pathology that involves the destruction of peri-radicular periodontal tissues as consequence of the persistent microbial infection of the root canal system and the host immune responses against it [131]. As periodontitis, AP is also a determinant cause of tooth loss and has been associated with low-grade systemic inflammation and the development of several non-communicable diseases, especially cardiovascular diseases [132]. The hallmark of this pathology is the formation of an osteolytic apical lesion of endodontic origin [56,90]. From a histopathological point of view, ALEOs will correspond either to periapical granulomas (PGs) or radicular cysts (RCs). The former consists of granulation tissue, which is highly rich in neo forming blood vessels [133]. 

Persistent root canal infection causes the release of pathogenic bacteria and/or their bioproducts into the periradicular tissues where periodontal ligament cells regulate the chemotaxis of infiltrating leukocytes [134], activating humoral and cellular responses. As a reaction to harmful exogenous elements, inflammation presents typical signs that include vasodilatation, uprising cellular metabolism, cellular influx, alteration of blood flow and extravasation of fluids [135]. 

VEGF has been detected in human PGs and RCs [65,136] at the connective tissue of both PGs and RCs and the epithelial lining of RCs [64,136]. This pattern of expression can be linked to wound healing, which coexists with inflammatory destruction during AP development. Furthermore, VEGF is also expressed in residual RC [136], defined as a RC which was not removed after the extraction of the causal infected tooth [137]. In fact, VEGF expression in residual RCs is lower compared to RC and PG [136], presumably because of the lack of remnant bacterial toxin stimulation. VEGF expression has also been associated with high MVD areas of both PGs and RCs [64], which reinforces its participation in neo vessel formation in AP. In ALEOs, VEGF could also contribute to the survival and maintenance of the capillary networks, as in vitro studies have determined that this growth factor prevents apoptosis of microvascular endothelial cells [138,139,140]. 

The activity and progression in AP are represented in two clinical stages of the disease; symptomatic AP and asymptomatic AP. The immune response is exacerbated in symptomatic AP, due to a loss of balance between the immune response and the bacterial infection of the root canal system [141]. Our study group has previously demonstrated that the expression of toll-like receptors (TLR)-2 and -4, which are involved in the recognition of damage and pathogen molecular patterns, were positively correlated with VEGF-A in human ALEOs. In the same study, mRNA levels of VEGF-A were detected in both, asymptomatic and symptomatic AP [67]. The detection of VEGF-A in both clinical entities is related to its role in endothelial cell proliferation associated with chronic inflammation, apparently without participation in the clinical exacerbation of the disease. Instead, the symptoms might be mediated by other cytokines and angiogenic factors, like IL-6, TNF-α and CDH5, which were significantly higher in symptomatic compared to asymptomatic AP [67]. 

Besides the above-mentioned in vitro study, in which VEGF soluble levels were significantly augmented by peroxide exposure and reduced by MMPs inhibition in hydrogen peroxide-stimulated human periodontal ligament fibroblasts [117], the relationship between MMP-9 and VEGF is further observed in human ALEOs. A strong expression of MMP-9 in human ALEOs has been previously associated with a significantly higher number of immunopositive cells for VEGF [142]. It has also been suggested that some inflammatory cells might be responsible for the synthesis and release of VEGF-A in early phases of AP [143], upregulating their multiple functions in inflamed tissues. 

The signaling pathways of VEGF in human ALEOs have also been studied [66]. VEGF-A-dependent pathologic angiogenic signaling pathways appear through the activation of protein kinase C-γ (PKC-γ) via Scr-dependent phospholipase D1 (PLD1) [144]. The activation of PKC-γ induces the proliferation and migration of microvascular endothelial cells and promotes the tube formation [144]. The PKC gene is upregulated in human ALEOs in comparison to healthy periodontal ligament (PDL), which indicates a higher endothelial activity among periapical inflammation [66]. In this study, a higher expression of phospholipase A (PLA2G6) and phosphoinositide 3-kinase (PIK3) genes were also demonstrated [66]. These genes are involved in pathologic angiogenesis and endothelial migration, respectively [145,146,147]. Other genes involved in endothelial cell interactions and activity are down regulated in human ALEOs. SHC adaptor protein 2 (SHC2), which is commonly expressed in endothelial cells and participates in VEGFR-2 activation, was down regulated in AP in comparison with healthy PDL samples [66]. Also, VEGFR-2 and RAC1 expression, involved in pathologic and physiologic angiogenesis [148], were lower in the ALEOs group [66]. Overall, in inflamed periapical tissues there is an alteration in the expression of several genes which may contribute to changes in endothelial cell activity and/or interactions. 

During inflammation lymphocytes, macrophages [149] and dendritic cells [150] exhibit the expression of VEGF-A, -C, -D and their receptors. This pattern is reproduced in immune cells and blood vessel endothelium in rat and human ALEOs [66,151]. The detection of VEGF and VEGFR in these cellular components of the immune system and their localization suggests that VEGFs may participate in the pathogenesis of ALEOs [66]. In vitro studies have demonstrated that VEGF promotes chemotaxis of immune cells [152,153], contributing to the extravasation of them to locally inflamed tissues. According to the localization of the receptors of VEGF in ALEOs it is also possible that immune cells functions take place through autocrine regulation of VEGFR-2 and -3 [66]. 

The expression of the VEGF family of cytokines and their receptors in osteoblasts and osteoclasts have been described, linking vascular growth and bone turnover [154,155]. Up to now their presence has been reported in experimentally-induced ALEOs in rats, particularly in osteoclasts where VEGFR-2 and -3 were also detected [151]. The signaling mechanisms of VEGF-A through VEGFR-1 are regarded to promote osteoclast differentiation [156]; whereas VEGFR-2 activation is associated with osteoclastic bone resorption [157]. The positive localization of VEGFR-2 in AP might explain one of the pathways responsible for loss of peri-radicular bone.

**Figure 2:**
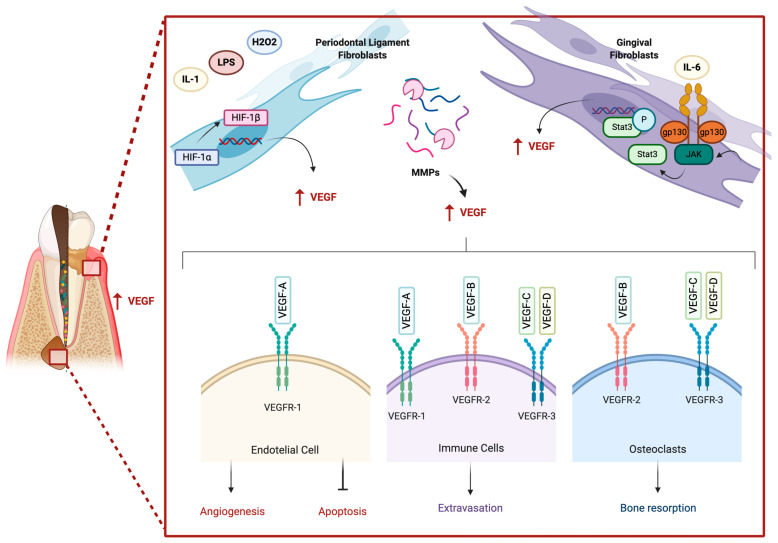
Role of VEGF in inflammatory periodontal diseases. During inflammatory periodontal diseases VEGF transcription can be induced by the activation of two pathways: (1) in periodontal fibroblasts IL-1, LPS and reactive oxygen species (H_2_O_2_) may induce the transcription of VEGF through the activation of the HIF/VEGF pathway. (2) The formation of the complex IL-6/IL-6R/gp130 may activate JAK/STAT pathway enhancing the secretion of VEGF by gingival fibroblasts. Also, VEGF might be released through the MMP-mediated proteolytic cleavage from its cryptic forms from the extracellular matrix. These cellular pathways might result in the following effects: VEGF in endothelial cells inhibits apoptosis and VEGF-A/VEGFR-1 interaction induces angiogenesis in immune cells, VEGFs participates in leukocytes’ extravasation and in osteoclasts VEGFR-2/-3 are associated with alveolar bone resorption. Created with Biorender.com.

A summary of the role of VEGF signaling in inflammatory periodontal diseases is presented in Figure 2.

### 3.3. Diagnostics

Nowadays, the diagnosis of periodontitis, peri-implantitis and AP are mainly based on the clinical history, signs, symptoms and imaging exams. Nevertheless, these mostly reflect the history of past disease, but show important pitfalls to reflect present disease, early diagnosis and predict future disease progression [158,159]. Thereby, biomarker identification in oral fluids represents a future goal to aid clinical diagnosis.

Saliva and GCF are both oral fluids acknowledged to harvest biological markers from periodontal/peri-implant tissues with high potential for biomarker-based diagnostics. Nevertheless, VEGF studies in saliva of periodontitis patients are still controversial, possibly because of the complex nature of saliva [57,58]. Noticeably, a recent study evaluated the VEGF levels in whole saliva and serum samples from periodontitis and healthy individuals, considering the smoking habit. In general, higher levels of VEGF were found in saliva and serum levels of periodontitis group, but when stratified, this difference was confirmed only for smokers. The authors reported a diagnostic precision of 0.88 for VEGF to discriminate periodontitis individuals from healthy controls, supporting its diagnostic potential [59].

GCF is a physiologic transudate or inflammatory exudate that reflects health and diseased periodontal status, respectively. Several studies have reported higher levels of VEGF in GCF from periodontitis patients in relation to healthy individuals [56,57,60,62,160,161] and reduction of its levels after conservative/non-surgical periodontal therapy [60,160,161]. However, no differences in its levels were found between periodontitis and healthy sites within the same periodontitis patients [61]. In an analogous manner to GCF, peri-implant crevicular fluid (PICF) reflects healthy and peri-implantitis tissues. Higher VEGF concentrations were detected in PICF from individuals with PI compared with clinically healthy implants, showing a strong correlation with pocket depth, suggesting that VEGF might participate in the progression of peri-implantitis [98]. In the same line, the combined detection of *Treponema denticola* together with IL-1β, TIMP-2 and VEGF were reported to strengthen the ability to diagnose peri-implantitis sites and also to have the potential to predict disease outcome [63]. Another multi-biomarker study reported that VEGF, IL-17 and TNF alpha in PICF were able to differentiate healthy sites of healthy implants from peri-implantitis sites from diseased implants with a diagnostic precision of 0.90. These outcomes propose that VEGF alone or combined with other biomarkers in GCF/PICF may aid to differentiate diseased sites [162]. 

Even though some investigations have proposed the analysis of the composition of GCF as a measurable method reflecting the presence and activity of ALEOs [163], up to now there is no available literature exploring whether oral fluids VEGF might serve as a biomarker in the diagnosis and prognosis of AP. A synthesis of the studies conducted in diagnostics of OSCC and inflammatory diseases affecting periodontal tissues is presented in Table 1.

### 3.4. Therapy and Projections

Given that periodontitis and peri-implantitis are chronic inflammatory pathologies characterized by bacterial triggering and the subsequent loss of attachment [164,165], mechanical debridement on the root or implant surface is the gold standard of periodontal and peri-implantitis therapies, respectively. This intervention is successful for the majority of patients; nevertheless, some patients with severe periodontitis do not respond well enough. Patients with refractory disease, characterized by low plaque scores and poor response to therapy [166], might be candidates for immune modulation therapy. In a similar manner to periodontitis and peri-implantitis, the main goal of AP therapy is to eradicate the infection and prevent microorganisms from infecting or re-infecting the perirradicular tissues [167]. Given its high success rate (~80%) [168], immunomodulatory therapy might be useful only in a small number of cases with persistent AP.

An in vitro study comparing periodontitis associated fibroblasts (PAF) with normal gingival fibroblasts demonstrated that VEGFR-1 mRNA was highly expressed in the former [169]. In the same study, the use of a specific VEGFR-1 inhibitor or its down regulation via RNAi, resulted in significantly increased tissue metalloproteinase inhibitors (TIMP)-1 and -2, and the subsequent decrease in the collagen breakdown [169]. These results suggest VEGFR-1 as a novel target to treat individuals with severe or refractory periodontitis [169]. Conversely, in vivo evidence suggests that anti-VEGF-A modulation has not favorable outcomes for periodontitis or peri-implantitis. A recent study in a rice rat model, which tends to naturally develop localized periodontitis without external intervention, demonstrated that the use of anti-VEGF-A monotherapy resulted in an extensive inflammatory response, distinguished by extreme alveolar bone loss and fibrosis in comparison with the non-monotherapy group [87]. Similarly, rat-specific anti-VEGF—mostly useful for the blockage of VEGF-A—hindered bone healing and implant osseointegration [88]. 

The blockage of VEGF receptors in AP has been explored in an experimental rat [170]. In this study, the use of antibodies against VEGFR-2 and -3 and the combination of them, demonstrated an anti-inflammatory effect of VEGFR-2 and a pro-inflammatory response to combined signaling of VEGFR-2 and -3 [170]. In humans, suppressors of cytokine signaling (SOCS) attenuate the gene expression of inflammatory and bone resorptive cytokines in periapical diseases, acting as natural blockers of particular inflammatory pathways [171]. Higher levels of SOC-1 and SOC-3 in human ALEOs showed a tendency to negatively correlate with their size [171]. SOC-3 has been associated with the blockage of Stat3 in the regulation pathway of VEGF [172]. 

Overall, VEGF might represent a therapeutic target candidate to interfere with the bone resorptive process, angiogenesis and immune response in periodontitis, peri-implantitis and AP. Nevertheless, based on its homeostatic and healing roles, which are also mediated by angiogenesis and migration/differentiation of osteoblastic progenitor cells [173,174], further studies are necessary to assess the plausibility and clinical outcomes of anti-VEGF therapy in these family of pathologies. A synthesis of the studies conducted in the therapeutics of OSCC inflammatory diseases affecting periodontal tissues is presented in Table 2.

## 4. Conclusions

It can be concluded that the VEGF/VEGFR pathway is an important angiogenic mechanism commonly overexpressed in oral cancer and inflammatory periodontal diseases. In oral malignancies, VEGF overexpression is associated with the development of nodal and distant metastases and poor outcome. In periodontitis, in addition of being associated with angiogenesis, it is also involved in the inflammatory and bone resorptive responses. VEGF has great potential to be used as diagnostic and prognostic biomarker, especially in oral cancer. However, more prospective multicenter studies are needed in order to validate its utility for these purposes. Finally, targeting angiogenesis as part of OSCC and periodontal inflammatory treatment might be a useful strategy, but more preclinic and clinical studies are needed before this can be translated into patient’s care.

## Figures and Tables

**Table 1:** VEGF in oral diagnostics of oral non-communicable diseases.

Author, Year	Study Design	Groups, *n*	Technique	VEGF Levels	*p*
Oral Cancer					
Sauter et al., 1999. [14]	Retrospective without follow-up	FFPE patient tissue samples from NOM (*n* = 10), MOD (*n* = 9), CIS (*n* = 6), stage I and II OSCCs (*n* = 9), stage III and IV OSCCs (*n* = 10).	NB, WB, IHC	VEGF is overexpressed at both protein and gene levels in OSCC cells lines and FFPE tissue samples compared to normal oral keratinocyte cell lines and NOM respectively.	<0.05
	In vitro	OSCC and other cell lines (*n* = 7).			
Nayak et al., 2012. [21]	Prospective with follow-up	Tissue biopsies and blood samples from PMOLs (*n* = 60), OSCC (*n* = 60) and healthy controls (*n* = 20).	IHC, qPCR, ELISA,	VEGF-A protein and gene expression were higher in PMOLs and OSCC compared to controls and higher in OSCC with node involvement than without.	<0.05
Seki et al., 2011. [22]	Retrospective with follow-up	FFPE patient tissue samples from well differentiated OSCCs (*n* = 72), moderately differentiated OSCCs (*n* = 12) and poorly differentiated OSCCs (*n* = 6).	IHC	Strong expression of VEGF-A or VEGF-C were effective prognostic predictors of OSCC survival.	N/A
Gandolfo et al., 2011. [20]	Retrospective without follow-up	FPPE patient tissue samples from OL with dysplasia (*n* = 18), OL without dysplasia (*n* = 11), OSCC (*n* = 40) and NOM (*n* = 20).	IHC	VEGF expression was higher in OL and OSCC than NOM and in OL with dysplasia than OL without dysplasia.	<0.05
López de Cicco et al., 2004. [24]	Retrospective without follow-up In vitro	FFPE patient tissue samples from ODs (*n* = 21), OSCCs, (*n* = 44), NOM (*n* = 46). Three SCC cell lines (SCC9, SCC15, SCC71).	IHC	All NOM samples presented with low or mild expression of VEGF-C, whereas strong expression was detected in 40% of ODs and 100% of OSCCs.	N/A
Aggarwal et al., 2014. [27]	Prospective without follow-up In vitro	Peripheral venous blood from patients with OSCC (*n* = 70) and healthy subjects (*n* = 30). Fresh OSCC biopsies (*n* = 17). OSCC cell lines (*n* = 4).	qPCR, WB, ELISA	VEGF expression was significantly upregulated in OSCC patients compared to healthy controls.	<0.05
				Serum VEGF levels were also higher in late stage tumors, large tumors and tumors with regional lymph node involvement. Treatment of OSCC cell lines with exogenous VEGF enhanced cell proliferation.	<0.05
Peterle et al., 2018. [28]	Retrospective with follow-up	FFPE patient tissue samples from OSCCs (*n* = 52).	IHC	Positive VEGF-A cytoplasmic expression was significantly associated with less differentiation tumor grade.	0.035
Faratzis et al., 2009. [29]	Retrospective with follow-up	FFPE patient tissue samples from TSCC (*n* = 87).	IHC	VEGF was overexpressed in 27.5% of all TSCC and correlated to the stage of the disease.	<0.05
				No prognostic significance of VEGF protein expression to survival status was found.	0.77
Shang et al., 2006. [30]	Retrospective without follow-up	FFPE patient tissue samples from OSCC (*n* = 40) and healthy controls (*n* = 20).	IHC, ELISA	VEGF positivity was correlated with regional lymph node involvement and tumor size.	0.004
Shao et al., 2008. [31]	Retrospective with follow-up	FFPE patient tissue samples from TSCCs (*n* = 59) and tumor free-oral mucosa (*n* = 10).	IHC	Higher expression of VEGF in TSCC compared to NOM.	0.01
				VEGF expression was an independent prognostic factor of overall survival was correlated with tumor size, clinical stage, lymph node invasion, recurrence and distant metastasis.	<0.05
				VEGF expression was an independent prognostic factor of overall survival.	<0.05
Morita et al., 2014. [32]	Retrospective with follow-up	FFPE patient tissue samples form TSCC (*n* = 40).	IHC	VEGF-C expression correlated with gender, tumor size, lymph node metastases, TNM and lymphatic vessel density.	<0.05
				Significant correlation between COX-2 and VEGF-C expression which was identified as an independent prognostic factor of overall survival.	<0.01
Arora et al., 2005. [25]	Retrospective with follow-up	FFPE patient tissue samples from OSCCs (*n* = 220), PL (hyperplasias = 59, dysplasias = 31) and matched normal oral tissues (*n* = 81).	IHC	VEGF was expressed in 76% of OSCCs, in 66% of PLs and in 25% of NOM. VEGF expression independently correlated with increased intratumoral microvessel density in PLs and OSCC. Increased VEGF expression was the most significant adverse prognosticator in OSCC patients.	N/A <0.05
Shintani et al., 2004. [33]	Retrospective without follow-up	FFPE patient tissue samples from OSCC (*n* = 98) and fresh OSCC specimens (*n* = 12).	IHC, WB, qPCR	60.2% of cases were positive for VEGF-A, 62.2% for VEGF-B, 67.3% for VEGF-C and 55.1% for VEGF-D	N/A
				VEGF-A and VEGF-B positively correlated with MVD and VEGF-C and VEGF-D expression were significantly associated with lymph node involvement.	<0.05
Lee et al., 2018. [38]	Retrospective with follow-up	FFPE patient tissue samples from hyperkeratosis (*n* = 8) and OSCCs (*n* = 30).	IHC	High VEGF expression in upper and lower epithelial layers had significant association in tumor metastasis and recurrence.	<0.001
Shang et al., 2007. [47]	Prospective without follow up	FFPE patient tissue samples from OSCCs (*n* = 31) and healthy controls (*n* = 10). Peripheral venous blood from OSCCs (*n* = 31) and healthy controls (*n* = 10).	ELISA, IHC	Mean VEGF level in OSCC patients (567.97 ± 338.17 pg.ml) was significantly higher than in normal controls (148.80 ± 64.17 pg.ml) and were positively correlated with metastasis and clinical stage.	<0.001
Kono et al., 2013. [46]	Retrospective with follow-up	FFPE patient tissue samples from OSCCs (*n* = 60).	IHC	VEGF-C levels were higher in metastatic tumors than non-metastatic and patient with high VEGF-C expression showed a shorter DSS tan patient with low expression.	<0.05
Patel et al., 2015. [49]	Prospective with follow-up	FFPE patient tissue samples from OSCCs (*n* = 109) and healthy controls (*n* = 50). Peripheral venous blood from OSCCs (*n* = 109) and healthy controls (*n* = 50).	ELISA, qPCR	Serum VEGF levels were significantly higher in patients with well differentiated recurrencies, large and advanced stage tumors. Patients having lower VEGF serum levels had significantly higher OS compared to patients with higher serum VEGF levels.	<0.05
Tse et al., 2007. [48]	Retrospective with follow-up	FFPE patient tissue samples from HNSCCs (*n* = 186).	IHC	Strong VEGF intensity was an independent adverse predictor for OS and DFS.	<0.05
Kazakydasan et al., 2017. [51]	Retrospective without follow-up	FFPE patient tissue samples from N0 OSCCs (*n* = 34).	IHC	High expression of VEGF-C in the primary tumor was associated with a greater probability for the occurrence of micrometastasis and isolated tumor cells in the lymph nodes.	0.011
Matsui et al., 2015. [52]	Retrospective with follow-up	FFPE patient tissue samples from TSCC (*n* = 90).	IHC	VEGF-C expression was associated with lymph node metastases and was a prognostic factor for DSS.	<0.05
Wakisaka et al., 2015. [53]	Retrospective with follow-up	FFPE patient tissue samples from OSCCs (*n* = 57).	IHC	Tumors with high VEGF-A and VEGF-D expression had significantly higher lymph vessel density than low expressing tumors.	<0.003
				VEGF-D expression was significantly higher in tumors with lymph node metastasis than in tumor without	<0.001
Al-Shareef et al., 2016. [55]	Retrospective with follow-up	FFPE patient tissue samples from TSCCs (*n* = 80).	IHC	VEGF-C and VEGFR-3 were not found independent predictor factors for lymph node metastasis of TSCCs.	>0.05
Naruse et al., 2015. [54]	Retrospective with follow-up	FFPE patient tissue samples from TSCCs (*n* = 65).	IHC	VEGF-C expression was associated with growth pattern and deep of invasion and VEGFR-3 expression was associated with growth pattern, pattern of invasion, deep of invasion and regional recurrences.	<0.05
				VEGF-C/VEGFR-3 expression was associated with regional recurrence, but was not identified as an independent factor for recurrence.	<0.05 >0.05
Inflammatory (periodontitis, periimplantitis, apical periodontitis)					
Booth et al., 1998. [56]	Cross-sectional	Gingival tissue, GCF and saliva from patients with periodontitis (*n* = 32) and healthy controls (*n* = 12).	IHC, ELISA	VEGF was detected in junctional, sulcular and gingival epithelium, neutrophils, macrophages and vascular endothelial cells. Some fibroblast was positive. No difference between VEGF levels in GCF between both groups. Higher levels of VEGF in saliva of patients with periodontitis in relation to controls.	<0.05
Afacan et al., 2019. [57]	Cross-sectional	GCF and saliva from G-AgP (*n* = 20), CP (*n* = 20), gingivitis individuals (*n* = 26) and healthy periodontal patients (*n* = 21).	ELISA	Higher total amounts of VEGF in GCF from G-AgP and CP groups than gingivitis and healthy groups, without difference between G-AgP and CP, whereas in saliva VEGF presented higher concentrations in gingivitis than healthy, CP and AgP groups.	<0.05
Sosnin et al., 2019. [58]	Cross-sectional	GCF and plasma from patients with generalized periodontitis (*n* = 42) and control healthy group (*n* = 36).	ELISA	No differences in the concentration of saliva and serum VEGF.	0.77
Şaştım et al., 2020. [59]	Cross-sectional	Saliva and serum from patients with periodontitis (20 smokers and 20 nonsmokers) and periodontally healthy controls (20 smokers and 18 nonsmokers).	Luminex	Higher concentrations of VEGF in saliva and serum in patients with periodontitis than periodontally healthy controls.	<0.001
				No difference in the concentrations of VEGF in saliva and serum between smokers and no smokers.	0.26
Romano et al., 2017. [60]	Cross-sectional	GCF from GAgP patients (*n* = 26) and healthy controls (*n* = 22).	Multiplex bead immunoassay	Higher concentrations of VEGF in from GAgP patients than healthy controls. Significant reduction of VEGF total amount after therapy in GAgP patients.	<0.01
A. Zekeridou et al., 2017. [61]	Cross-sectional	GCF from patients with chronic periodontitis (*n* = 24) and healthy controls (*n* = 20).	Bio-Plex suspension array system	No difference levels of VEGF among periodontitis site and healthy sites from periodontitis individuals and healthy sites from healthy control.	>0.05
Tayman et al., 2019. [62]	Cross-sectional	GCF from patients with generalized chronic periodontitis (*n* = 21), generalized aggressive periodontitis (*n* = 20) and healthy (*n* = 20).	ELISA	Highest total concentration of VEGF, followed by chronic periodontitis groups and lowest concentration in healthy controls.	<0.05
Wang et al., 2016. [63]	Cross-sectional	PICF from patients with peri-implantitis affected implant (*n* = 34) and healthy implant control (*n* = 34).	Human Quantibody arrays	Higher levels of VEGF in the peri-implantitis patients in relation to healthy implant control.	0.012
Graziani et al., 2006. [64]	Cross-sectional	RC (*n* = 24).	IHC	VEGF was detected in epithelial and connective tissues of RCs. Stromal cells showed higher levels of VEGF expression when compared with epithelial cells.	>0.05
Fonseca-Silva et al., 2012. [65]	Cross-sectional	RC (*n* = 40), PG (*n* = 28).	IHC	VEGF expression were similar in RC and PG.	>0.05
Virtej et al., 2013. [66]	Cross-sectional	ALEOs (*n* = 14) after endodontic surgery in patients diagnosed with CAP and PDL control samples (*n* = 4).	qPCR	Higher gene expression of VEGF-A and VEGFR-3 in ALEOS in comparison with PDL group.	>0.05
Fernandez et al., 2020. [67]	Cross-sectional	Symtomatic AP (*n* = 17), asymptomatic AP (*n* = 17).	qPCR	No difference in levels of VEGF-A mRNA.	>0.05

NOM: Normal oral mucosa, MOD: moderate oral displasia, CIS: carcinoma in situ, OSCC: Oral squamous cell carcinoma, HNSCC: head and neck squamous cel carcinoma, SCC: squamous cell carcinoma, OD: oral dysplasia, OL: oral leukoplakia, TSCC: tongue squamous cell carcinoma, NB: northern blot, WB: western blot, IHC: Immunohistochemistry, PMOL: potentially malignant oral lesion, PL: precancerous lesion, MVD: microvessel density, qPCR: quantitative PCR, N/A: not available., OS: overall survival, DFS: disease-free survival, DSS: disease-specific survival, GCF: gingival crevicular fluid, G-AgP: Generalized aggressive periodontitis, CP: chronic periodontitis, PICF: peri-implant crevicular fluid, PG: Periapical granulomas, RC: Radicular cysts, RRC: Residual radicular cysts, AP: Apical periodontitis, ALEO: Apical lesion of endodontic origin, CAP: apical periodontitis, PDL: periodontal ligament.

**Table 2:** VEGF pathway therapeutics in oral non-communicable diseases.

Author, Year	Study Design (RCT/Preclinical-Animal Model)	Intervention	Function	Outcome	*p*
Cancer					
Cohen et al., 2009. [76]	Phase I and phase II trials in patients with recurrent or metastatic HNSCC	Bevacizumab (15 mg/kg every 3 weeks) + erlotinib (150 mg/day)	Bvacizumab: anti-VEGF monoclonal antibody Erlotinib: EGFR inhibitor.	Response rate was of 15% with 4 complete responses (associated with expression of putative targets in pre-treatment tumor tissue).	N/A
Argiris et al., 2011. [77]	Phase II trial in patients with recurrent or metastatic HNSCC	Bevacizumab (15 mg/kg every 21 days) + Pemetrexed (500 mg/m^2^) every 21 days.	Bvacizumab: anti-VEGF monoclonal antibody Pemetrexed: multitargeted antifolate agent.	Overall response rate was of 30% (90% CI, 17–42%), disease control rate of 86% (90% CI, 77–96%) and there were 2 complete responses (5%).	N/A
Fury et al., 2016. [78]	Phase II trial in patients with stage III/IVB HNSCC	Bevacizumab (15 mg/kg) on day 1 and 22 + Cetuximab (400 mg/m^2^ on day minus 7 followed by weekly dosing of 250 mg/m^2^) + Cisplatin (two cycles of 50 mg/m^2^) with concurrent IMRT.	Bvacizumab: anti-VEGF monoclonal antibody Cetuximab: anti-EGFR chimeric monoclonal antibody.	The 2-year progression free survival was of 88.5% (95% CI, 68.1–96.1) and the 2-year overall survival of 92.8% (95% CI, 74.2–98.1%).	N/A
Argiris et a., 2019. [79]	Phase III RCT in patients with recurrent or metastatic HNSCC	Platinum based chemotherapy doublet with or without Bevacizumab (15 mg/kg every 3 weeks).	Cetuximab: anti-EGFR chimeric monoclonal antibody.	Median overall survival was 12.6 months for the group of chemotherapy + bevacizumab (BC) and of 11 months for chemotherapy alone.	0.22
				Median progression-free survival with BC was 6.0 months v 4.3 months with chemotherapy.	0.0014
				Overall response rates were of 35.5% with BC and 24.5% with chemotherapy.	0.016
Lalami et al., 2016. [83]	Phase II trial in recurrent or metastatic HNSCC	Sorafenib (1 cycle: 400 mg twice daily for 28 days).	Sorafenib: Multitarget small molecule inhibitor of wild-type and mutant B-Raf and c-Raf kinases, and tyrosine kinase domain of VEGFR-2,3 among others.	Only 1 patient had partial response (5%), 12 patients (55%) had stable disease and 9 patients (40%) had progressive disease. Early metabolic response rate was 38%.	N/A
Gilbert et al., 2015. [84]	Randomized phase II trial in recurrent or metastatic HNSCC	Cetuximab (400 mg/m^2^) on day 1 followed by 250 mg/m^2^ weekly) with or without Sorafenib (400 mg twice daily).	Cetuximab: anti-EGFR chimeric monoclonal antibody Sorafenib: Multitarget small molecule inhibitor of wild-type and mutant B-Raf and c-Raf kinases, and tyrosine kinase domain of VEGFR-2,3 among others.	Response rate was of 8% and clinical benefit of 12% for both groups. Median overall survival was 9 months in the cetuximab only group and 5.7 months in the combined group.	0.41
Zhou et al., 2010. [85]	Mouse 4-NQO model of oral carcinogenesis	Vandetanib (25 mg/Kg/day for 24 weeks) or nothing.	Vandetanib: Tyrosine kinase inhibitor with direct activity against multiple. Signal transduction pathways including VEGF-R2 and EGFR.	The use on Vandetanib reduced the occurrence of OSCC from 71% to 12% and of OD from 96% to 28%.	<0.001
Limaye et al., 2013. [86]	Randomized phase II trial in recurrent or metastatic HNSCC	Docetaxel (75 mg/m^2^ every 21 days) with or without vandetanib (100 mg, once a day).	Docetaxel: antimitotic chemotherapeutic Vandetanib: Tyrosine kinase inhibitor with direct activity against multiple signal transduction pathways including VEGF-R2 and EGFR.	Partial response was observed in 1 patient (*n* = 14) of the docetaxel only group and 2 patients (*n* = 15) of the combined group. Response rate and median overall survival were of 7% (95% CI, 0.2–33.8%) and 26.8 (95% CI, 17.7–100.7+) weeks in the single group and 13% (95% CI, 1.6–40.4%) and 24.1 (95% CI, 16.4–171.1+) weeks in the combined group.	N/A
Inflammatory (periodontitis, periimplantitis, apical periodontitis)					
Messer et al., 2020. [87]	Animal model	Anti-VEGF bvacizumab (5 mg B20-4.1.1/kg body weight, twice weekly.	Bvacizumab: anti-VEGF monoclonal antibody.	Rats developed mild to severe mandibular periodontitis.	N/A
Al Subaie et al., 2015. [88]	Animal model	Anti-VEGF, 4 ug diluted in 1.5 mL of saline, three times per week.	Anti-vascular endothelial growth factor neutralizing antibody: the blockage of VEGF-A.	Larger volume of the bone defects in the anti-VEGF rats in relation to controls.	0.026

HNSCC: head and neck squamous cell carcinoma, VEGF: vascular endothelial growth factor, EGFR: epithelial growth factor receptor, N/A: not available, CI: confidence interval, IMRT: intensity modulated radiation therapy, 4-NQO: 4-nitroquinolile 1-oxide, OSCC: oral squamous cell carcinoma, OD: oral dysplasia.

## Data Availability

Not applicable.
